# Supplementary material for: Imbalanced segregation of recombinant haplotypes in hybrid populations reveals inter- and intrachromosomal Dobzhansky-Muller incompatibilities
Source: PLoS Genet. 2022 Mar 28;18(3):e1010120. doi: 10.1371/journal.pgen.1010120 (PMC8989332; doi:10.1371/journal.pgen.1010120)
Supplement: Attachment — Submitted filename: 21-12-15_DMI-ms-response_final.pdf [file pgen.1010120.s036.pdf]

Response to Reviewers

Dear Editors,

Please find attached the revised version of our manuscript entitled “Imbalanced segregation of recombinant haplotypes in hybrid populations reveals inter- and intrachromosomal Dobzhansky-Muller incompatibilities” (previously “Inferring inter- and intrachromosomal Dobzhansky-Muller incompatibilities from imbalanced segregation of recombinant haplotypes in hybrid populations”) to be submitted to PLOS Genetics.

We thank you and the three reviewers for reviewing our paper and providing such detailed and constructive comments. We were delighted to see that the reviewers agreed that our method is promising for the application to the detection of epistasis. Due to the valuable and insightful comments, we believe that the revised version of our manuscript is much improved. We have carefully considered all comments and, specifically, performed more simulations to enlarge the tested parameter range. We extended the discussion of the limitations and possible extensions of the method. We showed that with even migration rates from two populations, DMIs can be detected after thousands of generations. Moreover, we added three-locus two-DMI models to generalize our statistics.

The details of the steps we took to address each point raised by the Editor and the Reviewers can be found in the response letter, highlighted in blue. In the manuscript, all changes are highlighted.

We hope that you find this version of the manuscript suitable for publication.

Thank you for your consideration.

Sincerely,

Claudia Bank,

on behalf of all authors

Reviewer #1:

Comments on “Inferring inter- and intrachromosomal Dobzhansky-Muller incompatibilities from imbalanced segregation of recombinant haplotypes in hybrid populations” by Li et al., submitted to Plos Genetics

This paper describes, evaluates, and applies two new statistics to detect intra- and interchromosomal DMIs in hybrids. The first statistic, X(2), detects deviation in the variance of two-locus heterozygosity from its expected value: X(2) < 0 indicates the presence of DMIs, especially when LD, measured as D’, is simultaneously negative. The second statistic, D2ik, describes the deviation in frequency of a specific two-locus haplotype relative to its expected frequency based on allele frequencies. It is additionally used to compute DeltaD2ik, the deviation in frequency between two haplotypes, for example between the two possible recombinant haplotypes. The statistics are evaluated deterministically and by stochastic simulations, and then applied to three hybrid populations of swordtail fish, where several DMIs could be detected.

I find the new statistics very interesting and a nice approach to detect epistasis. However, the paper tries to combine two topics: the method development on one side, and the results that come from its application to swordtails on the other side. As both topics are very complex, the results for either remain incomplete. I can thus imagine an improvement of the paper by focusing more on one or the other topic by reducing focus on the other.

Reply: We thank the reviewer for their positive evaluation of our manuscript, their many constructive suggestions and the detailed comments as well as spotting of typos. We agree that it was a challenge to combine the method development and the analysis in one paper while presenting the complete picture. Following the reviewers’ comments, we have extended the modelling and method development sections. We believe that this has resulted in a stronger manuscript that will reach a broader audience.

The method evaluations, specifically the simulations, were done with settings that explicitly fit the specifics of the swordtail system (e.g., lines 677-678). This is a good idea when the focus of the paper is on swordtails, but less so when the method should be applied to other systems. For example, few other systems will have population sizes of n=5000 or small migration rates <0.01 from the minor parent only. For being an empirical paper, on the other hand, the focus is too much on the methods and the conclusions that are currently drawn from the swordtail results are a bit limited.

Generally, I find it great to have methods like this developed, and it would be nice if other researchers could apply it to their data. For this it would be good though to clearly know about the strengths and limitations of the method under a wider range of realistic scenarios.

Reply: The reviewer is correct that our range of simulations was relatively limited. We have extended our analysis as follows. First, we have added simulations of small hybrid populations (population sizes from 300 to 5000, Figure 3A). Second (also following suggestions from reviewer 3), we have explored the $X(2)$ distributions in additional null models that include a large parameter space by combining migration rate and direct selection in our current version (Figure 6 and S14, see results, page 13). We show that $X(2)$ indicates recombinant imbalance across a large range of scenarios. To make the statistics and the method more accessible for other researchers, we have explained the deviation from the expected variance of heterozygosity more intuitively (Figure S3, see Results in page 3). Moreover, we have extended our discussion of the limitations and potential applications (see discussion “Detection time window strongly depends on population demography”).

Specific comments:

Simulations:
In case that the method should be applied more generally, the performance of the method would need to be assessed under a wider range of conditions.
- Population size: very large population sizes were simulated, which will be rather rare for most real hybrid populations. Is there a minimum population size required? How many samples are recommended to get good estimates for the two statistics?

Reply: Following this comment we have extended the parameter range of our simulations to population sizes as low as 300 (Figure 4A). Briefly, small hybrid populations (<1000) also allow us to detect the recombinant imbalance caused by DMI, but the time window is closed sooner due to genetic drift. Here at least one DMI locus tends to become monomorphic very quickly due to selection and drift (see results page 9).

The reviewer further inquired about the limits to the sample size for the applicability of our method. Our simulations showed that the minimum sampling size to get at least 50 pseudo-phased haplotypes for statistics is 200 (Figure S22, see Methods page 26). We strongly suggest sampling at least 300 individuals (see Figure 4A).

- Migration: Why only looking at minor-parent migration? What happens, when migration comes from both the minor and major parent: should not both considered together?

Reply: We thank the reviewer for suggesting to study also migration from both parental populations. With a continuous small influx from two parental populations, the DMI will persist for a very long time in the population (eventually persisting at migration-selection balance), theoretically creating an infinite window for the detection of imbalanced haplotype segregation. We added this scenario to Figure 2 and described it in the Results section (see page 8).

- Selection: I was wondering what other forms of selection might result in similar patterns (other than the tested Hill-Robertson interference); for example, stronger selection for either alpha or beta? Currently, alpha and beta were held nearly constant at very similar selection coefficients throughout the simulations (i.e., drawn from exponential distribution with mean 0.001, without separate evaluations).

Reply: Strong selection acting on one locus (such as at allele A) would simultaneously increase the frequencies of haplotypes AB & Ab without resulting in recombinant imbalance. Only strong epistatic effects (either positive or negative) and HRI like selection can create imbalance between recombinants. As we write in the revised version of the manuscript, “Without epistasis, only strong selection in the same direction acting on both *A* and *B* can generate recombinant imbalance by means of Hill-Robertson interference (Hill and Robertson 1966, Felsenstein 1974).”

- Recombination rate: Sensitivity and specificity were only estimated for c=0.5, when I understand this correctly? As the claim is that the method can detect both inter- and intrachromosomal DMIs, it would be good to explore performance for (a few) c<0.5 as well. In simulations (Tables S1-S3), DMI pairs were randomly distributed across chromosomes, but the effect of linkage on performance was not assessed separately (i.e., results were pooled across all possible linkages).

Reply: We agree that this is a valuable addition. We focused on c=0.5 in the previous version of the manuscript because it allowed the comparison with the G test. We have added results for simulations with c=0.1 in Figure S10, which are similar to the pattern in Figure 5 (see page 12). As highlighted with the deterministic toy model (Figure 2), reduced recombination leads to a shift of the detection window towards later generations.

Definitions:
- The paper focuses on DMIs, but as it is acknowledged in the concluding remarks, DMIs are only one kind of epistatic interactions – so the method could in fact detect any other epistatic interaction, including positive epistasis, not only hybrid incompatibilities. Is this correct? If so, it would be good to explicitly mention this at the beginning.

Reply: The reviewer is correct. We now mention and discuss this explicitly (see discussion "DMIs cause distinctive patterns of LD and variance in two-locus heterozygosity" on page 19):

"The conditions for negative $X(2)$ are met when the imbalance happens between a pair of repulsive haplotypes (here, ab and AB) and this pair of haplotypes is in deficiency (linkage disequilibrium $D = g_{ab} - p_a p_b < 0$)."

- There is a mismatch in the definition of fitness values in lines 102-103 vs. line 602

Reply: Thank you very much. We have corrected this mistake.

- Lines 661-662: "F1 individuals do not suffer from the DMI": this is an important point that needs to be mentioned much earlier in the manuscript, in my opinion

Reply: We have clarified this in both results and methods. It reads:

"Throughout this paper, we implemented "recessive" DMIs in our model by assuming that individuals that carry a single copy of the incompatible alleles do not suffer from reduced fitness." (page 8)

"Direct fitness is multiplicative and heterozygous-heterozygous individuals do not suffer from the DMI, similar to the "recessive" DMI scenario in Turelli and Orr 2000 and Blanckaert & Bank 2018" (page 25)

- I found the usage of the term 'homozygous' confusing. Does it mean homozygous within each parental population, but not necessarily in the hybrids? Or homozygosity in the classical sense, i.e., the same two alleles at a single locus in the hybrid population? The data were filtered to only contain homozygous loci for some of the analyses, but then the method should not be able to detect anything.

Reply: Thanks for pointing out this ambiguity. We have clarified it as "same ancestry state at one ancestry informative site" (in results see page 12) and explained also in the methods (see page 27).

- The terms 'migration' or 'migration from the minor parent' seem to be used as synonyms sometimes, please clarify

Reply: We have corrected this inconsistency.

Application to empirical data:

- It was not fully clear to me whether in the three replicate hybrid populations, migration comes solely from the minor parent, or whether there is additional migration from the major parent.

Reply: We apologize for the unclear formulation in the previous version of the manuscript. We clarified the demography of the hybrid populations in the revised version of the manuscript as follows: "All three hybrid populations are known to experience some level of ongoing migration from one parental population (CHAF, ~5%; TOTO, ~3%; TLMC, <<1%; Schumer et al. 2017 and Powell et al. 2020; Table S4)." (page 16; see also Table S4)

Other:

- It would be nice to see some example plots for interchromosomal interactions (simulations and empirical data), similar to the ones shown in Figure 3 and Figure S11

Reply: We are now providing such figures. Please see Figure S6 (simulated data; corresponds to Figure 3) and S19 (natural hybrid data; corresponds to Figure S11 in the previous version, which is now Figure S18).

- I was wondering whether it makes sense to have D2ik, and/or DeltaD2ik, be normalized to always fall within a specific range of values, independent on allele frequencies

Reply: This is an interesting suggestion. We currently are not aware of a suitable approach to normalize these statistics in a meaningful way, but this would be an interesting idea to follow up on in future studies.

Minor comments:

Lines 33-40: what is this class of methods (classical genetic mapping?), which is contrasted here to the approach in the next paragraph?

Reply: We are indeed referring to classical genetic mapping, and we have clarified this in the revised version of the manuscript (see page 2 line 49).

Line 71: Typo: double "that"

Reply: We have corrected this.

Lines 94-96: Can you provide a few more details why the statistic can distinguish the two sources of LD?

Reply: In the revised version of the manuscript, we have explained this in detail on page 3 line 107-109: "That is because with population structure, individuals breed locally, which increases the proportion of groups of homozygous individuals while maintaining polymorphism globally. This, in turn, increases the variance of heterozygosity as compared to the panmictic expectation." In addition, Figure S3 explained the scenario in DMIs (see results page 4 line 130-135).

Line 278: Typo: "X" missing from "(2)"

Reply: We have corrected this.

Lines 319-321: When I read the Figures correctly, sensitivity is reduced for the G test with

pseudo-phased data, but increased for the X(2) test (as in Figure S7, not S8, as referred to in the text)

Reply: Yes, that is correct. We now point this out in the manuscript on page 12 as follows:

“The performance trends for pseudo-phased data were similar to phased data with mildly increased sensitivities of $X(2)$ and decreased sensitivities of G test (Figure 5, S11 and S13).”

Line 423: sentence ends abruptly

Reply: We have corrected this as follows (see page 17 line 511):

“Altogether, these results strongly imply that a small set of loci is involved in complex DMI interactions in the hybrid population at a time point.”

Line 471: So far I can see it, negative X(2) are reported for CHAF and TOTO, but not all three hybrid populations

Reply: Yes, we note this as follows in the revised version of the manuscript (page 18):

“In CHAF and TOTO, markers linked to Xmrk passed the filtering criteria, but none in TLMC (Table S9)”

Figure 6: please indicate confidence intervals or standard deviations or anything like that

Reply: Thank you for your suggestion. We have used the Wald method; the results can be found in the revised Figures 5, S10, S11 and S13.

Figure S1B: must the selection coefficient for ‘ab’ in the HRI model not be 1?

Reply: That is correct. We have corrected this mistake.

Figure S8: Legend and caption do not match (black vs. red dots); panels A and B do not seem to show the same data in the range where the two zooms overlap

Reply: We thank the reviewer for pointing this out. We have updated the figure caption (now Fig. S12) to clarify why the data are not the same, and what is shown. It now reads: “Red dots indicate DMIs are observed before Generation 5 with a significant p value by the G test in panel A. Black dots indicate DMIs after Generation 5 in panel A and B. Only 9.9% of the simulations showed a significant p value by the G test ($p < 0.05$) in panel B.”

Figure S11 caption: only recombinant imbalance is shown in the Figure, not parental imbalance

Reply: We updated the Figure caption (now Figure S18) to correct this mistake.

Table S2 caption: I assume these are results for two DMIs, not a single DMI, as currently indicated?

Reply: This is correct. We have updated the caption accordingly.

Reviewer #2:

Article summary

Understanding the genetic basis of reproductive and hybrid fitness is a key aim of speciation genetics. In this article the authors present two novel (closely related) statistics, X(2) [main focus of the paper] and □D2, which can be used to detect both inter- and intrachromosomal candidate Dobzhansky-Muller incompatibilities (DMIs) from the frequency imbalance of recombinant haplotypes segregating in hybrid populations. Unlike commonly used methods based on linkage disequilibrium, the authors find that X(2) is specifically associated with recombinant imbalance caused by classical DMIs, and that it is less sensitive to population structure and residual linkage than alternative methods. Another novelty of X(2) is that it can be used to detect intrachromosomal DMIs without the need for a detailed recombination map.

The authors have simulated a broad range of scenarios to study the performance of their statistic. They find that the power of X(2) to infer DMIs depends on the recombination rate, admixture proportion, migration, the number of generations since hybridisation, and interactions between these factors. In comparison with the G test, an alternative method to detect DMIs, X(2) was shown to have high sensitivity much longer after hybridisation. The specificity of the method is very high for neutral loci unlinked to DMIs, but large windows around the causal DMI loci can show up as significant, making it more difficult to determine their exact location.

The method is most powerful when applied to phased data, but it can still be applied to unphased filtered data with some decrease in performance. It was great to see that the authors considered the performance of their method in this scenario as this makes it more widely applicable. For unphased, filtered data the proportion of significant pairs (false positives) decreases more slowly with distance from the DMI loci. The filtering can also inadvertently remove strong DMI loci that are nearly purged, such that only their flanking regions show the signature of the DMI.

The authors analyse three natural populations of swordtail fish hybrids between *Xiphophorus birchmanni* and *X. malinche* called CHAF, TLMC and TOTO. Thousands of putative DMIs were detected, and a small number of loci were involved in many of these (99.5% of the putative DMIs involved at least one locus involved in >10 interactions). 17 putative DMIs were shared between CHAF and TLMC, none between all three populations. 14 loci involved in putative DMIs were shared across all three populations of which four were previously identified. Whilst some putative intrachromosomal DMIs were detected, it was not possible to draw conclusions about the relative prevalence of inter- and intrachromosomal DMIs from this study.

My overall impression of the study is that it presents a valuable new tool to investigate the genetic basis of incompatibilities from haplotype data from hybrid populations, which overcomes some of the challenges such as residual linkage that affects alternative methods. The study neatly combines theoretical work with simulations and analysis of empirical data. The article is clearly written, and the conclusion acknowledges some of the limitations of the study in making firm statements about the relative importance of inter- and intrachromosomal DMIs. I recommend the article for publication with minor revisions because I felt that the haploid toy model presented is contradictory with the heterozygosity-based statistic proposed. However, I think this issue can be overcome with clarification (if these are not contradictory), or redoing some of the analysis using a diploid model previously published by some of the authors.

Reply: We thank the reviewer for her careful reading of our manuscript, her positive feedback, and the many constructive and detailed comments, which made it easy for us to incorporate them into the revised manuscript.

Major points

1. My main point of concern is that I fail to understand why the authors chose to present a *haploid* toy model, given that their main statistic of interest is based on two-locus *heterozygosity*. Furthermore, the data from natural swordtail hybrids they present is diploid, and they already have a diploid equivalent of the model published.

   It also seems that removing heterozygous loci for the simulation data has a non-negligible effect on the false positive rate, which might suggest that heterozygosity and the relative fitness of homozygous-homozygous, homozygous-heterozygous and heterozygote-heterozygote genotypes at the DMI loci affects the results.

   I would suggest that the authors either explain why they believe a haploid toy model to be appropriate, or that they replace the haploid model in the first part of the paper with a diploid version.

   Reply: We appreciate the reviewer's confusion about why we used a haploid model while we are proposing (and performing) an application of the statistics to diploid organisms. The reason is that considering a diploid model would significantly increase the number of parameters because both dominance of direct selective effects and dominance of epistasis need to be considered. The haploid model provides similar trajectories to diploid models with varying degrees of dominance, which allows us to use it as a minimal model to highlight the characteristics of our statistics without causing confusion (and additional complexity) over our choice of the dominance parameters. We now note this more clearly in the manuscript and we have added Figure 21 showing the X(2) trajectories for the haploid model as compared to diploid models with different dominance parameters.

2. I was impressed that authors did not just explore the performance of their method with simulations, but also applied their method to data from natural hybrid populations. This analysis of the swordtail hybrids did leave me with some questions. The authors report a very large number of putative DMIs (~1400-2200) involving 28-56% of the bins of markers. With the simulations they demonstrate that specificity is high for neutral unlinked markers, but that neutral markers linked to DMI loci can display negative X(2) values in a broad window around the causal locus. Nevertheless, in the simulated data the proportion of significant marker pairs decreases with distance from the DMI locus (Figure 7). This raises the question how the bins found to involve putative DMIs are distributed across the chromosomes in the swordtail hybrids and whether they also show consistent decreasing patterns that could be used to narrow down the location of the putative DMI. (Ideally this data could be provided as supplementary information). If that is not the case, does this suggest that the density of incompatibility loci is so high in this dataset that it obscures the signal of their location? Perhaps the authors could say a little more on how to proceed with such a vast number of DMI candidates.

   Reply: The reviewer raises a valid concern. To illustrate the distribution of DMI loci in the genome (also suggested by reviewer 3), we first connected neighbouring candidate 1Mb bins and then plotted all remaining interactions in a circular plot (Figure S17). Also after this procedure, putative DMI loci with multiple interactions (multi-hit) are standing out in the plot. We provided a list of loci with >10 interactions in Table S6. The pattern for multi-hit putative DMI loci is similar to the simulated data, where there tend to exist multiple interactions between true DMI loci and several of their flanking loci (Figure 3, S6, 7A and S15A). We summarized this as "Specifically, based on our simulations, we observe four phenomena

that generate negative X(2): true DMI interactions, interaction signals from a flanking region with a DMI locus, interaction signals from two flanking regions and interference between DMIs."(page 22).

We referred to DMI studies in other species to discuss the number of DMIs in the swordtail fish hybrid populations (see page 22): "However, we suspect that we can be confident in loci which we inferred to be involved in DMIs in multiple populations or interactions. Based on this, we speculate that we may have detected 50-70 loci involved in DMIs per swordtail fish hybrid population. In other species, at least 100 loci were experimentally identified among closely related species (reviewed in Wu and Palopoli 1994, Matute et al. 2010, Moyle and Nakazato 2010, Guerrero et al. 2017)."

3. A related point which the authors also address in the discussion (lines 550-555) is that the simulations and toy model with which the authors explore and test the behaviour and performance of their statistic consider one (or two) simple DMIs. Whilst this keeps the model tractable, the authors report that 99.5%. of candidate DMIs coming out of the analysis of the natural hybrid data involve a small number of interaction hub loci, each involved in >10 interactions. This raises the question whether we can extrapolate their results on pairwise, classical DMIs to the complex genetic architecture in this system, and whether this system is suitable to demonstrate the performance of the method. In fact, in their simulation results they found evidence for interference between DMIs (line 338 & 363). The authors even write that the loci that occur in only one or two putative DMIs may be considered as noise caused by true DMI loci, even though this is exactly the type of locus their method was designed to detect.

   It would be good if the authors could compare one additional simulation scenario with two DMIs that share one interaction partner, to see how the true and false positive rate compares to the one or two independent DMIs scenarios. This could help to understand what proportion of the large number of DMI candidates found might be expected if some loci are acting as interaction hubs and help to determine whether the implied assumption of treating DMIs as independent is justified.

   Reply: The reviewer raises a good point (although our simulations already contained a few complex interactions due to the random location of the DMI loci). We have now performed an additional set of simulations with shared interaction partners and included a discussion of these results in the manuscript (Figure 6 and S14, see results on page 13, and Method on page 26): "Notably, under the same selection and demographic parameters, when two DMIs share one locus (three-locus two-DMI model, see Methods), the $X(2)$ distribution at the DMI loci is slightly closer to zero than the distributions for two separate DMIs (Figure 6 and S14). In all cases, $X(2)$ distributions in both models can clearly separate DMI loci from neutral loci (Figure 6 and S14)."

Minor points

4. The abstract is well-written and appealing. My only suggestion would be to add that the signal that can be detected with the statistic proposed is transient e.g. 'we can detect DMIs up to XXX generations after hybridisation'.

   Reply: Thank you for the suggestion. Notably, with migration from both parental populations the signal can theoretically be detected for a very long time, as we show in the revised manuscript. We now write in the Abstract accordingly: "The detection window depends on the demographic scenario, the recombination rate, and the strength of epistasis."

5. For the model with two DMIs, I could not find how the fitness costs of each DMI were combined. I would suggest that the authors state this explicitly.

Reply: Thank you for suggesting this. In our simulations, the fitness is multiplicative, which is now clarified on page 26. It now reads: "Here, the fitness was computed as the product of the respective fitness of the two DMIs".

6. Due to a fairly large number of parameters, I understand it might not be possible to explore the parameter space more exhaustively, but it would be good if the authors could comment on how the predictions such as the timeframe of negative X(2) might be affected by changing the values of key parameters such as the selection coefficients or epistatic effect.

   Reply: Good suggestion. We have added this to the discussion "Detection time window strongly depends on population demography" accordingly (see page 20).

7. It looks like 1000 generations is insufficient to reach the peak in -X(2) for the simulations presented on the bottom row of figure 2. It would be preferred to run the simulations until the end of the time window of negative X(2) is reached.

   Reply: Following this comment we have now traced all trajectories for 10000 generations or until the DMI was lost (any allele frequency <0.005) from the populations. Notably, in the case of migration from both parental populations, migration-selection balance can be reached such that the DMI can persist in the population for an indefinite time (Figure 2 in the revised version of the manuscript).

8. I did not fully understand the statement on lines 403-404, 'the proportion of pairs of loci that fulfilled these criteria was similar to the proportion observed in simulations of one or two DMIs.' Could the authors clarify what they mean by this statement? I presume this is not a statement about the minimal number of DMIs required to explain the large number of significant pairs, but it might give that impression.

   Reply: Thank you for pointing out this unclear statement. This sentence was removed from the manuscript during the revision.

Line-by-line comments:

L39 'contributed' should be 'contributing'

Reply: We have corrected this.

L41 'Another commonly used approach' unclear what other approach this refers to

Reply: We have clarified that we are referring to classical genetic mapping (see page 2 line 49).

L65 'may expect DMIs within chromosomes may be more common' correct grammar

Reply: We have corrected this.

L84 'we find that DMIs…' should this be DMI candidates or putative DMIs?

Reply: We have updated this sentence.

L94 'X(2) …along the genome' can it be said to be along the genome given that it concerns pairs of loci?

Reply: Thank you for pointing this out. The sentence now reads: "Here, we demonstrate that a pairwise DMI, where n=2, changes $X(2)$ at DMI loci to negative values in a hybrid population, i.e., it creates a lower-than-expected variance in two-locus heterozygosity."

L146 ‘close to even’ could replace this with ‘similar’ to avoid confusion with ½.

Reply: Good suggestion. Thank you.

L174 ‘maximal’ should be ‘maximally negative’ or ‘most extreme’

Reply: We have changed “maximal” to “most extreme” accordingly.

L179 Could add citation of Hill & Roberston 1966

Reply: We have added two references. Hill & Roberston 1966 is the original paper, which focused on populations without recombination so that diffusion equations could be used, whereas Felsenstein 1974 implemented recombination.

L186 ‘lower maximum values’ could replace with ‘less extreme values’

Reply: We have changed the sentence accordingly.

L278 ‘(2) <’ should be ‘X(2) <’

Reply: We have changed the sentence accordingly.

L290 Legend mentions population size twice.

Reply: We have changed the legend accordingly.

L294 ‘DMI is present’ should this be ‘DMI locus is present’?

Reply: Thank you for pointing this out. We have corrected it.

L311 ‘no trade-off between sensitivity and specificity for X(2)’ is this also true for neutral loci on the DMI chromosomes when c is not 0.5?

Reply: Yes. We have added Figure S10 in which the recombination probability is 0.1.

L363 ‘interference between the two DMIs’ this is interesting in the light of the large number of putative DMIs in the swordtail analysis. It seems that interference or some loci being involved in many interactions is responsible for high false positive rates.

Reply: Thank you for this comment. We now explain on page 22: “Specifically, based on our simulations, we observe four phenomena that generate negative $X(2)$: true DMI interactions, interaction signals from a flanking region with a DMI locus, interaction signals from two flanking regions and interference between DMIs. However, we suspect that we can be confident in loci which we inferred to be involved in DMIs in multiple populations or interactions.”

L383 ‘Using’ should be in italics.

Reply: We have corrected it accordingly.

L395 ‘from the minor parental population’ I presume there is no migration from the other parental population?

Reply: We clarified the demography of the hybrid populations in the revised version of the manuscript as follows: “All three hybrid populations are known to experience some level of ongoing migration from one parental population (CHAF, ~5%; TOTO, ~3%; TLMC, <<1%;

Schumer et al. 2017 and Powell et al. 2020; Table S4). Under the demographic parameters of these natural populations, we expect that in CHAF and TOTO, immigration from minor parental population counterbalances the asymmetric admixture proportions such that we can infer DMIs with intermediate sensitivity and high specificity (similar to Figure 2, 5, S10 & S13)." (page 16; see also Table S4)

L404 Can the authors elaborate a bit on how many actual DMIs they expect there are in the natural hybrid populations from their analyses? Here it is suggested there could be just one or two causing this many significant pairs, but elsewhere (e.g. L84) they write that 'DMIs are widespread'.

Reply: We added a new paragraph to discuss the number of DMI loci between sibling species (see discussion page 22 line 696-708).

L407 Did these putative DMIs occur on all chromosomes? It would be great if this data could be provided as supplementary online.

Reply: We have presented the data as a supplementary figure (Figure S17) considering that a table would be very large.

L407-423 I found it slightly confusing that 'putative DMIs' and 'interactions' are used interchangeably, as well as 'bins' and 'loci', and 'interaction partners'. Might be helpful to clarify what is meant by locus, bin, marker, etc.

Reply: Thank you for pointing out this inconsistency. We now use the terms more specifically. We use "1Mb bin" to refer to a locus in the swordtail genome, we have replaced "interactions" by "putative DMIs", and we are referring to "locus" to point to the true DMI locus.

L423 Sentence incomplete, something missing?

Reply: We have corrected this sentence; it now reads: "Altogether, these results strongly imply that a small set of loci is involved in complex DMI interactions in the hybrid population at a time point." (see page 17).

L443 'hinting why' should be 'hinting at why'

Reply: We have corrected the sentence accordingly.

L453 'chr20:9M hybrid and chr20:20M' Is something missing here?

Reply: We have corrected this mistake, it now reads "between chr20:9M and chr20:10M".

L485 ΔD2 is not mentioned in the sections on the inference of putative DMIs.

Reply: We used ΔD2 to visually confirm recombinant imbalance in the intra- and inter-chromosomal DMIs (Figure S18 & S19). We now mention and discuss this more clearly in the Results (page 18). It reads:

"In the $\Delta D2$ heat plot of this genomic region, the candidate recombinant from TLMC (composed of *X. birchmanni* ancestry at chr20:9M and *X. malinche* ancestry at chr20:10M) also showed a weak signal of elimination in CHAF."

L529&537 ‘more prevalent’ and ‘slightly overrepresented’ The former seems to suggest >50% which is quite far from the 4% observed. Did the initial hypothesis take into account chromosome length?

Reply: Yes, we considered chromosome length. We have adjusted the sentence using “slightly overrepresented”.

L577 Moran et al. 2021 is missing from reference list.

Reply: We have added it.

L588 ‘Kryashimsky’ I think this should be spelled ‘Kryazhimskiy’

Reply: Thank you for pointing this out. We have corrected it accordingly.

L602 Fitness definition listed here differs from that in L103.

Reply: We have corrected this mistake.

L661 My understanding is that all double heterozygotes are assumed to be fit, ‘F1’ suggests that this would only apply to the first generation of hybrids.

Reply: You understood correctly. We corrected “F1 individuals” as “heterozygous-heterozygous individuals”.

L683 ‘Specificity was estimated as the proportion of DMIs that we failed to detect under a null model of neutral evolution.’ should be ‘that we incorrectly inferred’ or something along those lines.

Reply: Thank you for pointing this out. We have corrected it to “Specificity was estimated by the proportion of pairs that are correctly classified not to be DMIs under a null model of neutral evolution.”

L685 If the detection criteria are set such as to exclude false positives, then that sets specificity to 1. In Fig6, the deviation from 1 is due to noise?

Reply: Yes. In current version, it is Figure 5. We clarified in figure legend. It reads “To exclude sampling noise, any interaction with $X(2) < -0.005$ & $D' < 0$ was taken as a putative DMI.”

L696 ‘inter- or interchromosomal’ should be ‘inter- or intrachromosomal’

Reply: We have corrected it accordingly.

L746 Figure S11 is based on the swordtail data, not simulations as this sentence implies.

Reply: Yes. We marked the population name in the first row (Figure S18 and S19 in current version).

Figures:

F1 Legend does not explain white areas in the plots, perhaps copy explanation from Figure S4 legend.

Reply: Thanks for the suggestion; we have added an explanation as in Figure S5 (Figure S4 in previous version).s

F4 Does Panel A include non-hybrids?

Reply: Non-hybrids were excluded. We did not clarify this in previous version. In current version, we clarified this in the Methods (see page 25) and in the figure legend.

F6 I understand that the G test can only be applied to unlinked loci, but since the authors chose to present just the X(2) results in the main text, it would be good to see the increase in false positive rate for neutral loci linked to DMI loci reflected in this figure. Particularly as this is important to interpret the large number of putative DMIs found in the swordtail dataset.

Reply: Figure 7 and Figure S15 show how the number of significant interactions decreases with increasing distance from true DMI loci. Thus, neutral loci in flanking regions contribute to the significant interactions. However, it is difficult to decide whether to consider this signal as false positives.

F7 I find it a bit confusing that panel A also appears to have shades of blue, but as I understand it these do not have the same meaning as in panel B.

Reply: The color intensity has the same meaning in Panel A and B; it shows the strength of epistasis between the DMI loci, or, for flanking regions, the strength of epistasis of the nearest DMI.

Supplementary Figures:

S1 Size of arrows does not match selection coefficients. I also don't understand why haplotype ab has a selection coefficient of 1 under the DMI and 0 under the HRI model. Shouldn't these values be identical?

Reply: Thank you for pointing out this mistake. We have adapted the figure accordingly.

S3 I think the no DMI and strong DMI labels are swapped here.

Reply: (This comment refers to Fig. S4 in the revised version.) No, they are not swapped. Strong DMIs cause the recombinant imbalance (Figure S4 C&D). Without a DMI, two recombinant haplotypes are produced with the same probability, so no recombinant imbalance occurs, i.e., the two curves overlap (Figure S4 A&B).

S4 Typo in legend 'interreference' should be 'interference'

Reply: We have corrected the word accordingly.

S10 Would changing the number of loci involved in an interaction improve the fit?

Reply: That's a very interesting question. However, the number of possible models that reflect multi-locus interactions is so large that we decided to restrict our argumentation to pairwise interactions only.

S13 y-axis labels 'hapotypes' should be 'haplotypes'

Reply: We have corrected it accordingly.

Supplementary Tables:

S5 Columns labelled as 'percentage' are actually proportions

Reply: We have corrected it accordingly.

S6&S8 'Shcumer' should be 'Schumer'

Reply: We have corrected it accordingly.

Reviewer #3:

This study proposes two novel statistics for identifying targets of negative epistasis, such as BDMIs, from population genomic data. They propose two statistics, one based on the variance in two-locus heterozygosity and one based on haplotypic imbalance. They use two-locus simulations and forward population genetic simulations to explore the properties and performance of these statistics. Results indicate utility for detecting BDMIs when admixture is recent, and when admixture proportions are fairly even (which may be rare in nature), although the presence of gene flow from the minor population can somewhat ameliorate these limitations. While the temporal horizon of the method tends to be ephemeral, there is a a somewhat longer-lived signal when BDMIs are between loci on the same chromosome. The fairly limited parameter space where the authors' statistics have a signal tempers the impact of the study. But in general, this is high quality work with fairly strong novelty as well, and the paper is well-written. Hence, even if the applicability is somewhat limited, I think the study could merit consideration for PLOS Genetics. However, several key improvements are needed prior to publication, as detailed below.

Reply: We thank the reviewer for their feedback, the careful reading of the manuscript and their constructive comments.

Major comments:

1. I don't think think the authors have done a good enough job of putting their method in the context of other approaches. Essentially, the introduction now reads like "LD methods are bad, so we'll look for something better". This framing is problematic for a few reasons. * First, the authors overstate the problems with typical LD-based methods.
Line 47: "Though widely used, LD-based methods are known to have an unacceptably high false positive rate and low sensitivity, even when researchers attempt to account for confounders such as hybrid population demographic history (Schumer and Brandvain 2016, Satokangas et al. 2020)."
While advantageous to the present study's narrative, this is a pretty dramatic overstatement and not fully supported by the cited studies. The same goes for similar statements at the beginning of the Discussion. These statements should to be qualified to say that there can be high false positive rates in certain situations, such as for very recent admixture and ongoing gene flow between source populations (thus generating admixture LD, which has been recognized for some time).
* Second, LD is still an integral aspect of the authors' own study.
* Third and most importantly, there is actually a striking (but presently unacknowledged) complementarity between the authors' approach and typical disequilibrium-based incompatibility scans. The authors' method works well when admixture is recent and/or there is ongoing gene flow. Disequilibrium scans have elevated false positive rates in exactly those scenarios, and instead work best after the initial generations of admixture and without high ongoing levels of gene flow from source populations. The authors' approach is useful for intrachromosomal DMIs, whereas other approaches can struggle in this scenario. Framing the fit of the authors' method in this way would seem much more accurate and useful to the field.

Reply: We appreciate this comment and have rephrased our introduction and discussion accordingly (see page 2 line 62 and page 19 discussion section “Detection time window strongly depends on population demography”).

2. The authors need to more clearly identify and communicate the demographic parameter space in which their method is applicable. Overall, the authors have done a nice job exploring the impacts of a range of demographic factors on their statistics. However, a concern is that the demonstrated temporal horizon of the statistics is limited. Results from the toy model are mostly shown for just the first 150 generations after admixture, and the SLiM simulations use a nearly-best-case scenario of 50 generations. This all seems well and good if the methods are only intended for swordtail data. But otherwise, it seems like a key question for anyone considering using this approach is whether their population of interest fits into the potentially limited parameter space where the statistics are not merely negative, but produce negative values beyond those expected by chance from the background genomic distribution. My suggestion is that the authors should perform the analyses necessary to more fully express when their methods have power (especially with regard to admixture time), and to communicate the applicability of their statistics more clearly in the Discussion.

Reply: We have extended the range of tested parameters and demographic scenarios. X(2) can show that Long-term DMI (thousands) existing in a hybrid population from the simulation on migration from both parental populations (also suggested by reviewer 2, Figure 2D; see results section “Migration can narrow or widen the detection window”, page 8).

In addition, we reanalysed the condition for negative X(2). Under this condition, X(2) is not limited to the early phase of hybridization, but could be used at any population to detect the potential epistatic effects (Figure S3, see page 4).

We further discussed the applicability in the Discussion (see discussion section “Detection time window strongly depends on population demography”, page 20).

3. There is a need for greater statistical rigor in dealing with multiple testing and determining which outliers reject the null hypothesis.
The authors assume only 400 loci per chromosome in simulations. Real data may have dramatically more SNPs than that, even if pruned based on LD, and hence the multiple testing problem will explode. Relatedly:
Line 424: "Although our overall false positive rate is low, given the large number of tests performed in our evaluation of the real data, we can be most confident in putative DMIs that are detected in multiple populations."
The lack of statistical rigor here is concerning. I think it is the responsibility of the authors to use quantitative approaches to determine what is significantly unexpected under the null hypothesis. Deeper consideration of statistical threshold, multiple testing, and significance is needed.

***Reply:** Following the reviewer comments, we have expanded our analysis as follows:

a. To define the selection threshold, we first provide the negative X(2) distribution in four demographic scenarios (see Figure 6 and S14). We found that negative X(2) values caused the noise mainly clustered around 0. >>75% of negative X(2) values are >-0.005. At the same time, X(2) distributions of DMIs are shifted to more extreme negative values. This is the first reason to use X(2)<-0.005 (see page 13).

b. We then defined thresholds for the fish data (Table S5). “To increase our confidence in the candidates, we performed bootstrapping to ensure that the locus pair was consistently inferred to have negative $X(2)$ and $D' < 0$ (Table S5, see Methods). The

number of inferred DMIs is quite robust to different $X(2)$ criteria (from 0 to -0.015, Table S5). A putative DMI was identified if homozygous markers at this locus showed $X(2) < -0.005$ and $D' < 0$ (see also Methods), based on the observation that neutral and DMI loci can be separated by negative $X(2)$ at -0.005 from simulations (Figure 6 and S14).” This is the second reason use X(2)<-0.005 .

c. The overlap of inferred DMI pairs between populations is very small, with only 17 DMI pairs between TLMC and CHAF populations. In 400 bins (see Table S5), there are 400^2 potential interactions. The overlap between CHAF and TOTO is small. This is a bit lower than expected (28, binomial test, p= 0.036). The shared interactions are mainly contributed by two loci, chr15:15M (8 putative DMIs) and chr23:23M (8 putative DMIs). The small overlap might be due to the different demographic history.

4. Graphical presentation of the empirical analysis is absent from the main paper. I think the authors should find a way to bin their two-dimensional outliers into blocks, and then illustrate each of the resulting pairwise interactions on an image of the genome. This would offer a more intuitive depiction of the results than text and supplemental tables alone, or the limited supplemental figures current present. Such measures would enhance/demonstrate the value of the approaches for other potential users.

***Reply:** Thank you for suggesting this. We added a circular plot (Figure S17). Figure S17 is consistent with the occurrence spectra of putative DMI bins (Figure S16). The large number of putative DMIs (~1000) are induced by a small number of loci (60-70).

Minor comments:

Line 338: "When only homozygous loci (pseudo-phased data) were used for the inference, the true positive rate remained similar, but the false positive rate was elevated to 3.6-6.1%" Please clarify what's being done here and why the false positive rate goes up.

Reply: The false positive rate increases because of small sample size. We have added the following explanation on page 13: “This is because the reduction in sampling size due to pseudo-phasing stochastically results in extremely low numbers or absence of incompatible haplotypes.” (see also Figure 6, S14 and S22).

Line 402: Please clarify if "homozygous markers" are SNPs that appear to be fixed between source species, or otherwise how they were chosen. And please also clarify if you mean that only homozygous hybrid individuals were included for a given locus pair.

Reply: We have clarified it as “same ancestry state at an ancestry informative site” (in results see page 12) and explained also in the methods (see page 27).

Line 510: "Traditional methods of DMI detection are strongly affected by recombination rate variation"
This would be more accurate if "intrachromosomal" was inserted before "DMI".

Reply: We have changed the text accordingly.

Line 609: Perhaps just stick with either j or k for the allele at locus B.

Reply: We have changed the text accordingly.

Not being especially familiar with this literature, I found that a missing conceptual piece is the authors' specific meaning of two locus heterozygosity.

Line 611: "The expected heterozygosity at two loci is the sum of the heterozygosity at A and B"
What is precisely meant by "heterozygosity at two loci" in this usage? If pA and pB are each 0.5, then hA + hB is 1, even though double-homozygotes for any particular allelic combination would typically exist, and so this does not seem like an intuitive definition of two locus heterozygosity. Further explanation and citation would be helpful, in both the Methods and the Results (readers might go to either section first).

Reply: In response to the reviewer's comment, we incorporated a definition of the two-locus heterozygosity as "the sum of heterozygosity over two loci" in Nei and Roychoudhury 1974 (see page 4 and page 23).

Edits:

Line 34: perhaps "hundreds or thousands" would be more accurate.

Reply: We have changed the text accordingly.

Line 45: Corbett-Detig used RIL data from between-population crosses, not population genomic data.

Reply: Thank you for pointing this out. We have changed the text accordingly (see page 2 line 20).

Line 65: don't need "may" twice

Reply: We have rewritten this sentence, it now reads: "This genomic architecture of gene regulation suggests that DMIs within chromosomes are more common than DMIs between chromosomes from first principles."

Line 415: "numbers of partners" would be more illustrative than "numbers of occurrences"

Reply: Thanks for the suggestion, we have changed the text accordingly.

Line 423: unfinished sentence

Reply: We have rewritten this sentence, it now reads: "Altogether, these results strongly imply that a small set of loci is involved in complex DMI interactions in the hybrid population at a time point." (page 17).
