# Supplementary material for: Imbalanced segregation of recombinant haplotypes in hybrid populations reveals inter- and intrachromosomal Dobzhansky-Muller incompatibilities
Source: PLoS Genet. 2022 Mar 28;18(3):e1010120. doi: 10.1371/journal.pgen.1010120 (PMC8989332; doi:10.1371/journal.pgen.1010120)
Supplement: Attachment — Submitted filename: 20220211-DMI-response.pdf [file pgen.1010120.s037.pdf]

This revised manuscript has been evaluated again by the three original reviewers. Each notes and appreciates the improvements to the manuscript. Based on their close reading, the reviewers each make a modest set of suggestions for additional changes to the manuscript. I encourage the authors to consider these in preparing a final version of the manuscript.

We thank Dr. Alex Buerkle and Dr. Kristen Bomblies for the encouraging words and for handling our manuscript.

Reviewer #1: Comments on "Imbalanced segregation of recombinant haplotypes in hybrid populations reveals inter- and intrachromosomal Dobzhansky-Muller incompatibilities" by Li et al., re-submitted to Plos Genetics

This revised version of the manuscript has much improved to the former version and I'm very happy to see that all my previous comments have been thoroughly addressed. In particular, simulations were added to investigate the performance of the method under a wider range of demographic settings, such as small population size and different migration rates from both parental populations, and different recombination rates. In addition, previously unclear formulations have now been adjusted so that they are understandable/unambiguous, and errors have been corrected.
Overall, the manuscript is clear and well-prepared, and presents an important new method to the scientific community. As such I think that it will be a very valuable contribution to Plos Genetics. I have only a few very minor comments left.

We thank the reviewer for their positive evaluation of our revised manuscript.

Minor comments:

1)
Paragraph "Detection time window strongly depends on population demography" (lines 616-624): I very much like the discussion on the power and limitations of the approach. What I believe is still missing is the effect of dominance (Methods lines 791-793; Figure S21), which does not only affect the detection time window, but also the strength of the signal. I think that just one sentence mentioning this in the Discussion should be sufficient, so that readers will be aware of this limitation.
In Figure S21, it is unclear what h0<

*Reply: Good point. We added a sentence addressing this on page 20. Now it reads "Specifically, the time to expose the recombinant imbalance at a DMI increases with decreasing recombination rate between the DMI loci, weaker incompatibility, or weaker dominance of the DMI."

In Figure S21, we changed "h0<<h1" to "Homo-homo" to represent a DMI that is only expressed when both loci are homozygous.

2)
Figure 6 vs. Figure S14:
The captions are identical, except that one has a uniform distribution (-1, -0.001) and the other [-1, -

0.001). So far, it's only visible from the main text that the difference is that Fig. 6 shows phased, and S14 shows pseudo-phased data.

*Reply: We thank the reviewer for pointing out this error. The boundary was included during sampling, so it should be [-1, -0.001]. We have checked all parameters accordingly and corrected the text. We have marked all the panels generated from "pseudo-phased" data in Figure S14.

3)
Data: It needs to be mentioned where fish data (and potentially, simulation results) will be available. For fish data, this would ideally be the raw data and the curated data sets, i.e., phased and pseudo-phased data.

*Reply: We thank the reviewer for pointing out this omission. The data were published in Schumer et al. 2018 and Powell et al. 2020. We have added the Dryad links to the main manuscript and Table S4. Upon publication, we will also archive the GitHub repository that contains all annotated simulation and analysis code. This code also contains the processing steps to generate the pseudo-phased data.

Additional remarks:

4)
Lines 274-278: It now reads: "We first considered the effect of variable population size" and then "We further considered the effect of two demographic factors". Population size is a demographic factor, so this should be adjusted.

*Reply: We have adjusted the text accordingly (page 9).

5)
Line 739, remove "is"

*Reply: We have adjusted the text accordingly.

6)
Figure 5 vs. Figure S10:
It took me a little while to find the difference in the simulation settings presented in the two Figures: Fig. 5 shows the results for c=0.5, while Fig. S10 shows them for c=0.1. Perhaps make this clearer by rearranging the caption so that it does not feel like a "spot the difference" puzzle?

*Reply: Thank you for this suggestion. We have highlighted the recombination probability in the figure legend and, in S10, in the first sentence of the caption.

Reviewer #2: I am happy with the changes that the authors made to the manuscript and the additional simulation work they have done. They extended the parameter range of their simulations, which will be helpful for others who wish to apply the method to their own study system. Furthermore, the new results on migration from both parental populations reveals that their method might be able to detect DMIs across longer timeframes than the authors could demonstrate previously. The new simulations with 3-locus DMI pairs give some hints towards how the method behaves for complex DMIs, which seem to be prominent in the swordtail system. The new part of the discussion about the phenomena that generate

negative X(2) is also a valuable addition, which helps to interpret the swordtail data. It was nice to see the distribution of putative DMI loci from the swordtail data visualised in Figure S17, and I think perhaps this could be moved to the main text as there is currently no figure for this section.

Regarding my confusion about their choice of a haploid model, I appreciate the clarification they gave about the definition of their statistic in the methods section, and the new supplementary figure and explanation addressing different dominance scenarios.

The new Figure 6 does a good job at visualising the difference in X(2) values for the neutral and DMI(-linked) loci. I did not find the new Figure S3 particularly intuitive to interpret. It might help to explain what the green diagonal line is and to mark out impossible regions of the graph e.g. when frequency of ab is 1, the other frequencies cannot be >0.

Overall, I would recommend the paper for publication in PLOS Genetics.

*Reply: We thank the reviewer for her positive feedback and summary.

We appreciate the suggestion to move Figure S17 to the main text; however, since we already have 7 figures in the main text, we prefer to keep it in the supplement.

We have expanded the caption of Figure S3 to make it clearer.

Minor comments:
L172 – It looks like immigration from the parental population can either widen or narrow the detection window in Fig2B. For example, the box in the top right corner (6,287) to 96,55).

*Reply: We thank the reviewer for pointing this out. The immigration rate indeed has different effects depending on the choice of minor parental proportion. We have revised the text as follows: "Immigration from the parental population can narrow or widen the detection window.".

L201 – It would be helpful to add which panels in Fig2 we should look at/compare.

*Reply: It should be Figure 2 A&B. We have added instructions accordingly.

L385 – I think this should be -0.00025. Otherwise it would not make sense that X(2) values for neutral chromosomes are lower than for DMI chromosomes. I think perhaps the confusion came from ln vs log10 in Fig6?

*Reply: The value is -0.025. The maximum X(2) on neutral chromosomes is larger than -0.025. In Figure 6, >>75% of X(2) values on neutral chromosomes are larger than -0.005. For better readability (and responding to Reviewer 3's suggestion), we relabeled the y axis in the updated figures to show the non-transformed values.

L387 – close to 1 should be in brackets and there is a minus missing.

*Reply: We have corrected this mistake.

L409 – It might be good to mention the actual values for the true positive rate as well.

*Reply: We have added the values.

Fig6A – Does it make sense that X(2) is negative and also decreases over time for neutral chromosomes?

*Reply: Yes, it does. At the early stage of hybridization, the residual parental linkage leads to large positive X(2) values. Thus, most of X(2) values are larger than 0, and only a few X(2) fluctuate close to 0 or even to negative values. With time, alleles fluctuate away from the original admixture proportion by genetic drift. The variance of haplotype frequencies becomes larger, and the variance of X(2) becomes larger as well. For example, a haplotype composed of two minor alleles becomes rarer when the minor allele frequencies become smaller with increasing hybridization age. At small sampling sizes, stochastic absence of this haplotype would lead to negative X(2).

L889 – 'makers' should be 'markers'

*Reply: We have corrected this typo.

L1141 – 'A&C. Sensitivity is high unless strong migration leads to swamping of the DMI alleles.' In FigS10C the lines without migration seem to show the poorest sensitivity?

*Reply: We have clarified this statement as follows: "Trajectories ended (indicated by large plot markers) when one of the interacting loci became monomorphic (A&B) or when there were less than 50 homozygous genotypes available to compute the statistics (C&D)." in Figure S10-S13.

Supp Figures - It might be helpful to emphasize the differences between Fig S10, S11 and S13, perhaps using titles on the figures.

*Reply: We thank the reviewer for this suggestion. We have added informative titles to each panel.

Code availability – Perhaps the authors could also provide the link to the original swordtail dataset, which I think is on NCBI?

*Reply: We have added the Dryad links to the published date in Table S4 and the main text.

Reviewer #3: I sympathize that the authors needed to respond to a large number of reviewer requests overall. They have definitely improved their manuscript. However, they are still only part-way there in terms of addressing my major comments:

1. I suggested that there was unacknowledged complementarity between the useful parameter space of the authors' approach and that of typical LD-based approaches. The revised text still does not speak to this complementarity, in that there is no acknowledgement of any parameter space where conventional LD approaches have high power and low false positive rates (i.e. histories where admixture is not very recent,

and there is not a high level of ongoing gene flow). I am troubled by the persistent tendency to disparage a set of approaches that may still be our best option for many taxa.

*Reply: We thank the reviewer for clarifying their point of concern. We have now acknowledged this point in discussion (page 20) as follows:

"Using the $X(2)$ statistics could be an excellent complementary approach to DMI detection by more established methods. For example, conventional LD-based methods to detect DMIs have moderate power and low false positive rates when admixture did not occur in the last tens of generations and when there is little ongoing gene flow, but they are vulnerable to high false positive rates when there is migration from parental populations (Schumer and Brandvain 2016, Satokangas et al. 2020). Moreover, they are limited to the inference of interchromosomal DMIs in the absence of accurate recombination maps."

2. I said "The authors need to more clearly identify and communicate the demographic parameter space in which their method is applicable..." The authors have somewhat broadened the set of demographic scenarios in their temporal simulations. Although they don't really address whether a predicted negative value of their metric is strong enough for rigorous BDMI detection, this section is still good enough for me if significance is adequately addressed further down.
A minor note - Figure 2C could be clearer in terms of which population gives/receives migrants.

*Reply: We have added the requested information in the Discussion (page 20-21) as follows:

"In this paper, we have shown that $X(2)$ reliably detects DMIs at the exact position at which the DMI is located. However, we also show that the signal of the DMI loci propagates into their flanking regions. In this vein, Blanckaert and Payseur 2021 discussed the hitchhiking effect that is caused by strong DMIs. Even if DMI loci are already monomorphic or at low frequency beyond the detection limit, linked polymorphic loci could still carry the signal of recombinant imbalance. Here we classify this case as a false positive, but in practice considering linked variation could extend the time window for DMI detection. In the future, it would be desirable to extend our method to include such linkage information, i.e., from multiple pairs of negative $X(2)$ between two genomic blocks, which could allow for more confident inference of DMI candidates."

In Fig. 2C, we have changed "Immigration from one parental population" to "Immigrat. from the par. pop. on x-axis" to be clearer.

3. I asked for "greater statistical rigor in dealing with multiple testing and determining which outliers reject the null hypothesis". The authors have made some progress in this area, including by illustrating the distribution of X(2) under neutral and DMI scenarios (Figure 6), and from this analysis they choose a cutoff of $X(2) < -0.005$ to heuristically define a positive result. However, little has changed in their empirical analysis, and it's still not clear to me whether the "false discovery rate" indicated in the Methods incorporates any consideration of genome-wide multiple testing, in order to yield a meaningful true/false positive probability. If not, then to get around linkage effects, perhaps it would be worth considering how many unique pairs of chromosomes had at least one window pair with X(2) below a cutoff, compared with how many chromosome pairs we'd expect to meet that threshold under the null hypothesis.

*Reply:

We appreciate the suggestion of the reviewer to compare the number of interchromosomal "hits" with a null distribution. However, defining the null hypothesis is not straightforward. The simulated data suggest that X(2) has a low false positive rate on neutral chromosome in all 400 simulations considering four demographic scenarios (Figure 6) when X(2)<-0.005. However, in the presence of DMIs, the false positive pairs increased to ~3% of the total tests (Table S2) and 6% for pseudo-phased data (Table S3).. Here, the total number of tests was: $\binom{400}{2}$ tests per simulation, 100 simulations for four-locus two-DMI model; $\binom{300}{2}$ tests per simulation, 100 simulations for three-locus two-DMI model. Most false positives occur due to linkage to DMIs. Thus, they still indicate a true DMI locus nearby, and in future work, we will address this in more detail. In addition, there is some background noise that is not related to a nearby DMI locus. Regarding the real data, there likely exist incompatibilities on every chromosome, so we would not have a "null set" of chromosomes to develop a baseline expectation of the X(2) cutoff. Moreover, even if we had a pair of chromosomes without DMIs from real data to compose a null hypothesis, it would not translate into a reasonable null hypothesis for intrachromosomal DMIs, where linkage plays a strong role for the X(2) dynamics at DMI loci and in their vicinity.

Regarding our chosen approach, we apologize that the details of our bootstrapping approach for determining an FDR were still not clear in the previous version of the manuscript. We have expanded its explanation as follows to clarify (page 28):

"The proportion of putative DMIs in all pairwise comparisons was very similar to the expected false positive rate obtained from our simulations, and the proportion of putative DMIs was elevated when markers were assigned to 1Mb bins (Table S3 and S5). To be conservative, given this result, we used several approaches to gain confidence in our DMI candidates. We first bootstrapped 20 times for each thinned dataset. Each bootstrap sample contained 700 markers. We used the median proportion p_{bin} of candidate bins with negative $X(2)$ over the 20 bootstrap samples as a baseline false positive rate. We then computed the probability of sampling the same candidate interaction between bins n times (based on choosing different markers in each round); this number should be binomially distributed with parameter p_{bin}. Using a binomial test, we determined k as the least number of times that an interaction candidate should be observed to be retained as a putative DMI. For this, we used a p-value threshold corrected for an arbitrarily determined false discovery rate of 0.001 (following the Benjamini-Hochberg procedure, Table S5), such that the p-value is smaller than (# of putative DMI pairs at the p-value threshold)/(total number of significant pairs in 20 bootstrap samples) · 0.001. We obtained values of k between 6 and 10 for the three hybrid populations (see Table S5) when $X(5) < -0.005$. As an additional approach to reduce false positives, we also limited our focus to interactions that were observed in multiple populations. Independent observation of interactions in two or more populations provides strong evidence that these interactions are not expected by chance."

Minor notes related to this point:
* In Figure 6, the quantity "-log(-X(2))" is challenging to intuit. I wonder about just plotting X(2) itself on a log-transformed y-axis.

*Reply: We thank the reviewer for this suggestion, which we have incorporated in Figure 6.

* Further discussion of the number of loci per chromosome would also be helpful. It seems to boil down to a question of the optimal window size in light of the number of generations since admixture.

*Reply: We have added some additional sentences addressing this issue to the Discussion on page 21: "Since the ancestry tract lengths are negatively correlated with the duration since the onset of hybridization (e.g., Ungerer et al. 1998), young hybrid populations are then expected to yield strong signals of DMIs across large genomic blocks, whereas older hybrid populations are expected to yield weaker but possibly more finely located DMI signals."

My original minor comments have been satisfactorily addressed, so it is basically points 1 and 3 above where I still have concerns.
