# Supplementary material for: Imbalanced segregation of recombinant haplotypes in hybrid populations reveals inter- and intrachromosomal Dobzhansky-Muller incompatibilities
Source: PLoS Genet. 2022 Mar 28;18(3):e1010120. doi: 10.1371/journal.pgen.1010120 (PMC8989332; doi:10.1371/journal.pgen.1010120)
Supplement: Attachment — Submitted filename: Review_Li_2021_PLoSgenetics_final.docx [file pgen.1010120.s035.docx]

**Article summary**

Understanding the genetic basis of reproductive and hybrid fitness is a key aim of speciation genetics. In this article the authors present two novel (closely related) statistics, X(2) [main focus of the paper] and ΔD2, which can be used to detect both inter- and intrachromosomal candidate Dobzhansky-Muller incompatibilities (DMIs) from the frequency imbalance of recombinant haplotypes segregating in hybrid populations. Unlike commonly used methods based on linkage disequilibrium, the authors find that X(2) is specifically associated with recombinant imbalance caused by classical DMIs, and that it is less sensitive to population structure and residual linkage than alternative methods. Another novelty of X(2) is that it can be used to detect intrachromosomal DMIs without the need for a detailed recombination map.

The authors have simulated a broad range of scenarios to study the performance of their statistic. They find that the power of X(2) to infer DMIs depends on the recombination rate, admixture proportion, migration, the number of generations since hybridisation, and interactions between these factors. In comparison with the G test, an alternative method to detect DMIs, X(2) was shown to have high sensitivity much longer after hybridisation. The specificity of the method is very high for neutral loci unlinked to DMIs, but large windows around the causal DMI loci can show up as significant, making it more difficult to determine their exact location.

The method is most powerful when applied to phased data, but it can still be applied to unphased filtered data with some decrease in performance. It was great to see that the authors considered the performance of their method in this scenario as this makes it more widely applicable. For unphased, filtered data the proportion of significant pairs (false positives) decreases more slowly with distance from the DMI loci. The filtering can also inadvertently remove strong DMI loci that are nearly purged, such that only their flanking regions show the signature of the DMI.

The authors analyse three natural populations of swordtail fish hybrids between *Xiphophorus birchmanni* and *X. malinche* called CHAF, TLMC and TOTO. Thousands of putative DMIs were detected, and a small number of loci were involved in many of these (99.5% of the putative DMIs involved at least one locus involved in >10 interactions). 17 putative DMIs were shared between CHAF and TLMC, none between all three populations. 14 loci involved in putative DMIs were shared across all three populations of which four were previously identified. Whilst some putative intrachromosomal DMIs were detected, it was not possible to draw conclusions about the relative prevalence of inter- and intrachromosomal DMIs from this study.

My overall impression of the study is that it presents a valuable new tool to investigate the genetic basis of incompatibilities from haplotype data from hybrid populations, which overcomes some of the challenges such as residual linkage that affects alternative methods. The study neatly combines theoretical work with simulations and analysis of empirical data. The article is clearly written, and the conclusion acknowledges some of the limitations of the study in making firm statements about the relative importance of inter- and intrachromosomal DMIs. I recommend the article for publication with minor revisions because I felt that the haploid toy model presented is contradictory with the heterozygosity-based statistic proposed. However, I think this issue can be overcome with clarification (if these are not contradictory), or redoing some of the analysis using a diploid model previously published by some of the authors.

**Major points**

1. My main point of concern is that I fail to understand why the authors chose to present a *haploid* toy model, given that their main statistic of interest is based on two-locus *heterozygosity*. Furthermore, the data from natural swordtail hybrids they present is diploid, and they already have a diploid equivalent of the model published.

It also seems that removing heterozygous loci for the simulation data has a non-negligible effect on the false positive rate, which might suggest that heterozygosity and the relative fitness of homozygous-homozygous, homozygous-heterozygous and heterozygote-heterozygote genotypes at the DMI loci affects the results.

I would suggest that the authors either explain why they believe a haploid toy model to be appropriate, or that they replace the haploid model in the first part of the paper with a diploid version.

2. I was impressed that authors did not just explore the performance of their method with simulations, but also applied their method to data from natural hybrid populations. This analysis of the swordtail hybrids did leave me with some questions. The authors report a very large number of putative DMIs (~1400-2200) involving 28-56% of the bins of markers. With the simulations they demonstrate that specificity is high for neutral unlinked markers, but that neutral markers linked to DMI loci can display negative X(2) values in a broad window around the causal locus. Nevertheless, in the simulated data the proportion of significant marker pairs decreases with distance from the DMI locus (Figure 7). This raises the question how the bins found to involve putative DMIs are distributed across the chromosomes in the swordtail hybrids and whether they also show consistent decreasing patterns that could be used to narrow down the location of the putative DMI. (Ideally this data could be provided as supplementary information). If that is not the case, does this suggest that the density of incompatibility loci is so high in this dataset that it obscures the signal of their location? Perhaps the authors could say a little more on how to proceed with such a vast number of DMI candidates.
3. A related point which the authors also address in the discussion (lines 550-555) is that the simulations and toy model with which the authors explore and test the behaviour and performance of their statistic consider one (or two) simple DMIs. Whilst this keeps the model tractable, the authors report that 99.5%. of candidate DMIs coming out of the analysis of the natural hybrid data involve a small number of interaction hub loci, each involved in >10 interactions. This raises the question whether we can extrapolate their results on pairwise, classical DMIs to the complex genetic architecture in this system, and whether this system is suitable to demonstrate the performance of the method. In fact, in their simulation results they found evidence for interference between DMIs (line 338 & 363). The authors even write that the loci that occur in only one or two putative DMIs may be considered as noise caused by true DMI loci, even though this is exactly the type of locus their method was designed to detect.

It would be good if the authors could compare one additional simulation scenario with two DMIs that share one interaction partner, to see how the true and false positive rate compares to the one or two independent DMIs scenarios. This could help to understand what proportion of the large number of DMI candidates found might be expected if some loci are acting as interaction hubs, and help to determine whether the implied assumption of treating DMIs as independent is justified.

**Minor points**

4. The abstract is well-written and appealing. My only suggestion would be to add that the signal that can be detected with the statistic proposed is transient e.g. ‘we can detect DMIs up to XXX generations after hybridisation’.
5. For the model with two DMIs, I could not find how the fitness costs of each DMI were combined. I would suggest that the authors state this explicitly.
6. Due to a fairly large number of parameters, I understand it might not be possible to explore the parameter space more exhaustively, but it would be good if the authors could comment on how the predictions such as the timeframe of negative X(2) might be affected by changing the values of key parameters such as the selection coefficients or epistatic effect.
7. It looks like 1000 generations is insufficient to reach the peak in -X(2) for the simulations presented on the bottom row of figure 2. It would be preferred to run the simulations until the end of the time window of negative X(2) is reached.
8. I did not fully understand the statement on lines 403-404, ‘the proportion of pairs of loci that fulfilled these criteria was similar to the proportion observed in simulations of one or two DMIs.’ Could the authors clarify what they mean by this statement? I presume this is not a statement about the minimal number of DMIs required to explain the large number of significant pairs, but it might give that impression.

**Line-by-line comments:**

L39	‘contributed’ should be ‘contributing’

L41	‘Another commonly used approach’ unclear what other approach this refers to

L65	‘may expect DMIs within chromosomes may be more common’ correct grammar

L84	‘we find that DMIs…’ should this be DMI candidates or putative DMIs?

L94	‘X(2) …along the genome’ can it be said to be along the genome given that it	concerns pairs of loci?

L146	‘close to even’ could replace this with ‘similar’ to avoid confusion with ½.

L174	‘maximal’ should be ‘maximally negative’ or ‘most extreme’

L179	Could add citation of Hill & Roberston 1966

L186	‘lower maximum values’ could replace with ‘less extreme values’

L278	‘(2) <’ should be ‘X(2) <’

L290	Legend mentions population size twice.

L294	‘DMI is present’ should this be ‘DMI locus is present’?

L311	‘no trade-off between sensitivity and specificity for X(2)’ is this also true for	neutral loci on the DMI chromosomes when c is not 0.5?

L363	‘interference between the two DMIs’ this is interesting in the light of the

large number of putative DMIs in the swordtail analysis. It seems that interference or some loci being involved in many interactions is responsible for high false positive rates.

L383	‘Using’ should be in italics.

L395	‘from the minor parental population’ I presume there is no migration from the other parental population?

L404	Can the authors elaborate a bit on how many actual DMIs they expect there are in the natural hybrid populations from their analyses? Here it is suggested there could be just one or two causing this many significant pairs, but elsewhere (e.g. L84) they write that ‘DMIs are widespread’.

L407	Did these putative DMIs occur on all chromosomes? It would be great if this data could be provided as supplementary online.

L407-423	I found it slightly confusing that ‘putative DMIs’ and ‘interactions’ are used interchangeably, as well as ‘bins’ and ‘loci’, and ‘interaction partners’. Might be helpful to clarify what is meant by locus, bin, marker, etc.

L423	Sentence incomplete, something missing?

L443	‘hinting why’ should be ‘hinting at why’

L453	‘chr20:9M hybrid and chr20:20M’ Is something missing here?

L485	ΔD2 is not mentioned in the sections on the inference of putative DMIs.

L529&537	‘more prevalent’ and ‘slightly overrepresented’ The former seems to suggest >50% which is quite far from the 4% observed. Did the initial hypothesis take into account chromosome length?

L577	Moran et al. 2021 is missing from reference list.

L588	‘Kryashimsky’ I think this should be spelled ‘Kryazhimskiy’

L602	Fitness definition listed here differs from that in L103.

L661	My understanding is that all double heterozygotes are assumed to be fit, ‘F1’ suggests that this would only apply to the first generation of hybrids.

L683	‘Specificity was estimated as the proportion of DMIs that we failed to detect under a null model of neutral evolution.’ should be ‘that we incorrectly inferred’ or something along those lines.

L685	If the detection criteria are set such as to exclude false positives, then that sets specificity to 1. In Fig6, the deviation from 1 is due to noise?

L696	‘inter- or interchromosomal’ should be ‘inter- or intrachromosomal’

L746	Figure S11 is based on the swordtail data, not simulations as this sentence implies.

**Figures:**

F1	Legend does not explain white areas in the plots, perhaps copy explanation from Figure S4 legend.

F4	Does Panel A include non-hybrids?

F6	I understand that the G test can only be applied to unlinked loci, but since the authors chose to present just the X(2) results in the main text, it would be good to see the increase in false positive rate for neutral loci linked to DMI loci reflected in this figure. Particularly as this is important to interpret the large number of putative DMIs found in the swordtail dataset.

F7	I find it a bit confusing that panel A also appears to have shades of blue, but as I understand it these do not have the same meaning as in panel B.

**Supplementary Figures:**

S1	Size of arrows does not match selection coefficients. I also don’t understand why haplotype ab has a selection coefficient of 1 under the DMI and 0 under the HRI model. Shouldn’t these values be identical?

S3	I think the no DMI and strong DMI labels are swapped here.

S4	Typo in legend ‘interreference’ should be ‘interference’

S10	Would changing the number of loci involved in an interaction improve the fit?

S13	y-axis labels ‘hapotypes’ should be ‘haplotypes’

**Supplementary Tables:**

S5	Columns labelled as ‘percentage’ are actually proportions

S6&S8	‘Shcumer’ should be ‘Schumer’
